# Aging, climate change, and the paradox of preparedness: Reflections from Villa El Salvador, Lima, Peru

**DOI:** 10.1371/journal.pgph.0006600

**Published:** 2026-06-12

**Authors:** Arman Majidulla, Oscar Flores-Flores

**Affiliations:** 1 Department of International Health, Johns Hopkins Bloomberg School of Public Health, Baltimore, Maryland, United States of America; 2 Centro de Investigación del Envejecimiento, Facultad de Medicina Humana, Universidad de San Martín de Porres, Lima, Peru; PLOS: Public Library of Science, UNITED STATES OF AMERICA

Across Latin America, adults over 65 faced a 1000% increase in heatwave exposure days in 2025 compared to three decades ago, and heat-related mortality in the region has more than doubled [1–3]. These figures show governments, funders, and international agencies the scale of the crisis, and why it demands attention and resources. But these measures favour what is most readily counted: deaths, acute illness, discrete heat events. The slower, cumulative harms of climate change are far harder to register. They cannot easily count Dora *(name and details fictionalized)*, an older woman in Villa El Salvador, a district shaped by rapid urbanization and uneven access to basic services, who cannot fill her water tank because she lives alone and nobody told her the water was running. Climate change reaches her not as a heatwave but as an accumulation of everyday gaps in services and support.

A growing and consequential body of literature has established older adults as a priority population at the intersection of aging and climate change. The Lancet Countdown tracks heat-related mortality among those over 65 as a sentinel indicator [3]; the WHO’s Decade of Healthy Ageing acknowledges environmental determinants of healthy aging [4]; and the broader literature carefully articulates how cognitive decline, frailty, social isolation, and poverty compound older adults’ exposure to climate risk [5]. In low-resource urban settings in the Global South, economic pressures reshape family and community care. Families migrate, work longer hours, and juggle informal employment, leaving older adults with less support while often increasing the responsibilities they must assume. At the same time, governments and institutions fail to provide the social and physical infrastructure that meaningful adaptation requires.

Villa El Salvador sits on the southern periphery of Lima, Peru, built by migrant families who constructed a community without much state investment - it was founded in 1971 when thousands of displaced families organized a mass settlement on unoccupied land [6]. It remains a district where infrastructure is uneven, housing is precarious, and formal support systems are difficult to access. Today, many older adults living there confront a changing climate that erodes their autonomy and adaptive capacity while offering little government support. Their situation is not simply about age. It reflects a longer history: communities that contributed least to climate change have received the fewest resources to respond to it. Displacement and structural neglect shaped these conditions long before the current crisis.

In November 2025, we convened an informal community meeting with ten community health workers (CHWs) living and working in Villa El Salvador, as part of the VIDACTIVA project, a community-based mental health initiative supporting older adults through goal-setting and social engagement [7,8]. Most of the CHWs were themselves older adults and long-term residents of the district. They spoke from both experience supporting participants and personal familiarity with the local context. The meeting was exploratory, intended to surface how those closest to older adults in this community understood the relationship between a changing climate and daily life. What emerged was not a catalogue of dramatic weather events but a description of accumulated difficulty: pavements that become impassable after unexpected rain, and disruptions to water supply that shift the burden of household water security onto individuals. Families stretched thin by economic necessity, and digital health systems that excluded those without the literacy or assistance to use them. It became clear that, for older adults, climate change does not arrive mainly as an acute event. Instead, it gradually narrows what they can safely do in everyday life, just as their ability to adapt is declining. Heat mortality statistics cannot capture these shifts. They count deaths, but they miss the slow constraints that shape the lives leading up to them.

The experiences described in Villa El Salvador district are not exceptional. They reflect a broader reality facing older adults across low-resource urban settings in the Global South, where the social and physical foundations for climate adaptation did not disappear suddenly but reduced over time. Vaughn’s [9] concept of engineered vulnerability helps explain this pattern: communities that bear the greatest burden of climate change are often those whose vulnerability was shaped by displacement and the sustained withdrawal of state investment. Older adults are not passive, older adults adapt [10]. But as Adger and colleagues [11] argue, the limits of adaptation lie within society itself. Such limits emerge from the values, relationships, and infrastructure that structure daily life. When these foundations are weak or absent, shifting responsibility onto individuals is not only insufficient; it misplaces accountability.

Recognition of the value of CHWs in Peru should evolve into sustained institutional support that strengthens and sustains their contributions to community health. In this context, older CHWs may be particularly well positioned to work with older adults because of shared lived experiences, stronger peer connection, and a deeper understanding of aging-related challenges. Second, greater efforts toward sustainable support mechanisms, including financial compensation, ongoing training, referral pathways, and supportive supervision, could help reduce the burden of relying exclusively on voluntary work. Third, creating opportunities that promote social cohesion among older adults, such as community spaces, communication networks, and collective intergenerational activities, may help strengthen participation and social support. Finally, CHWs could play a more formal role as liaisons between older adults, local services, and municipal structures, helping communicate community needs while also facilitating accountability and coordination across sectors. Without stronger institutional support, the contributions of CHWs may remain undervalued, underutilized, and difficult to sustain in the long term.
